# Supplementary material for: Systematic epigenome editing captures the context-dependent instructive function of chromatin modifications
Source: Nat Genet. 2024 May 9;56(6):1168–80. doi: 10.1038/s41588-024-01706-w (PMC11176084; doi:10.1038/s41588-024-01706-w)
Supplement: Supplementary file 1 — Reporting Summary [file 41588_2024_1706_MOESM1_ESM.pdf]

nature portfolio

Corresponding author(s): Jamie Hackett

Last updated by author(s): 09.01.2024

Reporting Summary

Nature Portfolio wishes to improve the reproducibility of the work that we publish. This form provides structure for consistency and transparency in reporting. For further information on Nature Portfolio policies, see our Editorial Policies and the Editorial Policy Checklist.

Statistics

For all statistical analyses, confirm that the following items are present in the figure legend, table legend, main text, or Methods section.

n/a	Confirmed	
☐	☒	The exact sample size (*n*) for each experimental group/condition, given as a discrete number and unit of measurement
☐	☒	A statement on whether measurements were taken from distinct samples or whether the same sample was measured repeatedly
☐	☒	The statistical test(s) used AND whether they are one- or two-sided<br>*Only common tests should be described solely by name; describe more complex techniques in the Methods section.*
☒	☐	A description of all covariates tested
☐	☒	A description of any assumptions or corrections, such as tests of normality and adjustment for multiple comparisons
☐	☒	A full description of the statistical parameters including central tendency (e.g. means) or other basic estimates (e.g. regression coefficient) AND variation (e.g. standard deviation) or associated estimates of uncertainty (e.g. confidence intervals)
☐	☒	For null hypothesis testing, the test statistic (e.g. *F*, *t*, *r*) with confidence intervals, effect sizes, degrees of freedom and *P* value noted<br>*Give P values as exact values whenever suitable.*
☒	☐	For Bayesian analysis, information on the choice of priors and Markov chain Monte Carlo settings
☒	☐	For hierarchical and complex designs, identification of the appropriate level for tests and full reporting of outcomes
☒	☐	Estimates of effect sizes (e.g. Cohen's *d*, Pearson's *r*), indicating how they were calculated

Our web collection on statistics for biologists contains articles on many of the points above.

Software and code

Policy information about availability of computer code

Data collection

Flow cytometry data was collected using the Attune NxT Flow Cytometer (Thermo Fisher Scientific). Sequencing data was collected using the Nextseq Illumina sequencing system. qPCR data was collected using QuantStudio5 (Applied Biosystems) thermal cycler. Pyrosequencing was run on PyroMark Q24 advanced pyrosequencer (Qiagen). Cell images were taken using the Leica Application Suite X (v3.5.7.23225). No custom software was used in this study.

Data analysis

Data analysis was performed using Graphpad Prism version 8.4.3 graphical software, FlowJo (v10.5.3), PyroMark Q24 Advanced 3.0 software, the Galaxy workflow interface maintained by EMBL Genome Biology Computational Support (v 23.0.6.dev0), R statistical software (v3.6.2) using Bioconductor packages, and with Seqmonk (v1.47.0) mapped sequence data anaylser.

RNAseq: Raw Fastq reads were trimmed to remove adaptors with TrimGalore (0.4.3.1, -phred33– quality 20–stringency 1 -e 0.1–length 20), quality checked and aligned to the mouse mm10 (GRCm38) genome using RNA Star (2.5.2b-0, default parameters except for–outFilterMultimapNmax 1000). Analysis of the mapped sequences was performed using Seqmonk software (Babraham bioinformatics, v1.47.0) to generate log2 reads per million (RPM) or gene length-adjusted (reads per kilobase million, RPKM) gene expression values. Differentially expressed genes (DEG) were determined using the DESeq2 package (v.1.24.0), inputting raw strand-specific mapping counts and applying a multiple-testing adjusted (FDR) P< 0.05 significance threshold, and log2 fold-change filter where indicated.

CUT&RUNseq: Raw Fastq sequences were trimmed to remove adaptors with TrimGalore (v0.4.3.1, -phred33 --quality 20 --stringency 1 -e 0.1 --length 20), quality checked and aligned to the mouse mm10 genome with the inserted mCherry reporter using Bowtie2 (v2.3.4.2, -I 50 -X 800 --fr -N 0 -L 22 -i ‘S,1,1.15’ --n-ceil ‘L,0,0.15’ --dpad 15 --gbar 4 --end-to-end -- score-min ‘L,-0.6,-0.6’). Analysis of the mapped sequences was performed using Seqmonk software (Babraham bioinformatics, v1.47.0) by enrichment quantification of the normalised reads. To identify

promoters with H3K4me3 change in Mll2CM/CM, a 1kb window centered on the TSS was quantified amongst replicates and a normalised log fold-change (FC) filter applied between samples. Metaplots over genomic features were constructed by quantifying 100bp bins centered on the features of interest and normalised cumulative enrichments plotted.

ATACseq: Following sequencing, raw reads were first trimmed with TrimGalore (v0.4.3.1, reads > 20 bp and quality > 30) and then quality checked with FastQC (v0.72). The resulting reads were aligned to custom mouse mm10 genome containing the reporter using Bowtie2 (v2.3.4.3, paired-end settings, fragment size 0-1,000, --fr, allow mate dovetailing). Aligned sequences were then analysed with seqmonk (Babraham bioinformatics, v1.47.0) by performing enrichment quantification of the normalised reads.

For manuscripts utilizing custom algorithms or software that are central to the research but not yet described in published literature, software must be made available to editors and reviewers. We strongly encourage code deposition in a community repository (e.g. GitHub). See the Nature Portfolio guidelines for submitting code & software for further information.

Data

Policy information about availability of data

All manuscripts must include a data availability statement. This statement should provide the following information, where applicable:

- Accession codes, unique identifiers, or web links for publicly available datasets
- A description of any restrictions on data availability
- For clinical datasets or third party data, please ensure that the statement adheres to our policy

Manuscript includes a data availability statement. All data derived from next generation sequencing assays have been deposited in the publically available ArrayExpress database under the accession codes E-MTAB-12101, E-MTAB-13466, E-MTAB-13467, E-MTAB-13468.
https://www.ebi.ac.uk/biostudies/arrayexpress/studies/E-MTAB-12101
https://www.ebi.ac.uk/biostudies/arrayexpress/studies/E-MTAB-13466
https://www.ebi.ac.uk/biostudies/arrayexpress/studies/E-MTAB-13467
https://www.ebi.ac.uk/biostudies/arrayexpress/studies/E-MTAB-13468
Additionally, previously published ChiP-seq data from Nora et al. 2017 is used in this study: GSE98671, https://www.ncbi.nlm.nih.gov/geo/query/acc.cgi?acc=GSE98671
All data is publicly available.

Human research participants

Policy information about studies involving human research participants and Sex and Gender in Research.

Reporting on sex and gender	N/A
Population characteristics	N/A
Recruitment	N/A
Ethics oversight	N/A

Note that full information on the approval of the study protocol must also be provided in the manuscript.

Field-specific reporting

Please select the one below that is the best fit for your research. If you are not sure, read the appropriate sections before making your selection.

☒ Life sciences ☐ Behavioural & social sciences ☐ Ecological, evolutionary & environmental sciences

For a reference copy of the document with all sections, see nature.com/documents/nr-reporting-summary-flat.pdf

Life sciences study design

All studies must disclose on these points even when the disclosure is negative.

Sample size	Information on sample size is provided within each figure legend. Sample sizes were based on prior research experience for similar assays rather than power analysis. Biological replicates are defined as measurements of biologically distinct samples (e.g. independently derived clonal reporter lines each separately transected with epigenetic editing machinery; or independently (and freshly) derived genetically-modified ESC lines from blastocysts). Technical replicates are defined as repeated measurements of the same sample that show independent measures of the noise associated with the equipment and the protocols.
Data exclusions	To generate the heat maps shown in figures 4C and 6A, typically normalised geometric mean values coming from four technical replicates of the experiments were averaged and log2 transformed. Log2 fold-change values were plotted in R. In rare cases, outliers identified as extremely different values relative to all other values within the dataset were excluded. Data exclusion was also performed whenever cross-contamination between different samples was suspected.<br>No data was excluded from RNAseq, CUT&RUNseq, CUT&RUN-qPCR and bisulfite pyrosequencing experiments.

Replication

All reported findings are reliably reproducible. The impact of epigenetic marks on transcription in different genomic contexts was validated using different reporter clones across at least three independent experiments and by performing repeated measurements of the same samples over time. Reproducibility between independent RNAseq and CUT&RUNseq samples was assessed on binned and library-normalised files using multiple clustering approaches including PCA, correlation assessment, and unsupervised hierarchal clustering in R and Seqmonk software, with good reproducibility observed. All experiments with quantification data were repeated at least three times in biologically independent experiments (the only exception being epigenetic mark deposition assessment for catalytic mutant effectors in fig. 2c-k for which in some cases n = 2 biological replicates). All replications were successful, with coherent results.

Randomization

No randomization of data was performed as the study does not involve a clinical trial, human subjects or mice. All cell lines within each experimental paradigm/experiment were cultured concurrently to minimise batch variability which is not the result of treatment or genetic background.

Blinding

The investigators were not blinded during experiments because no subjectivity in assessment of experimental results was possible in our study. Indeed all data collection and quantification was performed via dedicated and quantitative instruments (Attune NxT Flow Cytometer, qPCR thermal cycler etc..) therefore avoiding biased outcomes. Additionally all results were checked and interpreted by two different individuals.

Reporting for specific materials, systems and methods

We require information from authors about some types of materials, experimental systems and methods used in many studies. Here, indicate whether each material, system or method listed is relevant to your study. If you are not sure if a list item applies to your research, read the appropriate section before selecting a response.

Materials & experimental systems

n/a	Involved in the study
☐	☒ Antibodies
☐	☒ Eukaryotic cell lines
☒	☐ Palaeontology and archaeology
☒	☐ Animals and other organisms
☒	☐ Clinical data
☒	☐ Dual use research of concern

Methods

n/a	Involved in the study
☐	☒ ChIP-seq
☐	☒ Flow cytometry
☒	☐ MRI-based neuroimaging

Antibodies

Antibodies used

Rabbit anti-H3K4me3 (Diagenode Cat#C15410003), Rabbit anti-H3K27me3 (Millipore Cat#07-449), Rabbit anti-H3K9me3 (Abcam Cat#ab8898), Rabbit anti-H3K9me2 (Active Motif Cat#39041), Rabbit anti-H2Aub (Lys119) (CST Cat#8240), Rabbit anti-H3K36me3 (Diagenode Cat#C15410192), Rabbit anti-H3K36me3 (Active Motif Cat#61101), Rabbit anti-H3K27ac (Active Motif Cat#39133), Rabbit anti-H3K79me2 (Abcam Cat#ab3594), Rabbit anti-H4K20me3 (Abcam, Cat#ab9053)

Validation

All used antibodies are commercially available and have been validated by the manufacturer. Validations and detail product information are available on the manufacturer's websites.

Rabbit anti-H3K4me3 (Diagenode Cat#C15410003): the manufacturer has validated the antibody specificity by ChiP using the iDeal ChiP-seq kit and has determined the specificity by dot blot performed with peptides containing other histone modifications and the unmodified H3K4. The manufacturer states: "Figure 5A shows a high specificity of the antibody for the modification of interest".
https://www.diagenode.com/en/p/h3k4me3-polyclonal-antibody-premium-50-ug-50-ul

Rabbit anti-H3K27me3 (Millipore Cat#07-449): the manufacturer has validated the antibody specificity by dot blot. The manufacturer states: "1 μg/mL of this antibody detected Trimethyl-Histone H3 (Lys27), but not unmethylated Histone H3 (Lys27) or other peptides corresponding to modified histones in an Absurance™ Histone H3 Antibody Specificity Array (Cat. No. 16-667) and in an Absurance™ Histone H2A, H2B, H4 Antibody Specificity Array (Cat. No. 16-665)".
https://www.merckmillipore.com/IT/it/product/Anti-trimethyl-Histone-H3-Lys27-Antibody,MM_NF-07-449

Rabbit anti-H3K9me3 (Abcam Cat#ab8898): the manufacturer has validated the antibody specificity by western blot. The manufacturer states: "Histone H3 (tri methyl K9) antibody (ab8898) is specific for Histone H3 tri methyl Lysine 9. Shows slight cross-reactivity with tri methyl K27, which shares a similar epitope (please see Western blot image). Does not react with mono or di methylated K9".
https://doc.abcam.com/datasheets/histone-h3-tri-methyl-k9-antibody-chip-grade-ab8898.pdf

Rabbit anti-H3K9me2 (Active Motif Cat#39041): the manufacturer has validated the antibody specificity by dot blot and peptide array analysis.
https://www.activemotif.com/catalog/details/39239/histone-h3-dimethyl-lys9-antibody-pab

Rabbit anti-H2Aub (Lys119) (CST Cat#8240): the manufacturer states: "Ubiquityl-Histone H2A (Lys119) (D27C4) XP® Rabbit mAb recognizes endogenous levels of histone H2A protein only when ubiquitinated at Lys119. The antibody does not cross-react with other ubiquitinated proteins or free ubiquitin".
https://www.cellsignal.com/products/primary-antibodies/ubiquityl-histone-h2a-lys119-d27c4-xp-rabbit-mab/8240

Rabbit anti-H3K36me3 (Diagenode Cat#C15410192): the manufacturer has validated the antibody specificity by dot blot and by peptide array analysis. The manufacturer states: "Figure 5A shows a high specificity of the antibody for the modification of interest. The peptide array analysis shows a slight cross reaction with H4K20me3 that was not observed in dot blot".
https://www.diagenode.com/en/p/h3k36me3-polyclonal-antibody-premium-50-mg

Rabbit anti-H3K36me3 (Active Motif Cat#61101): Dot blot analysis was used to confirm the specificity of Histone H3K36me3 antibody for trimethyl-lysine 36 of histone H3.
https://www.activemotif.com/catalog/details/61101/histone-h3-trimethyl-lys36-antibody-pab

Rabbit anti-H3K27ac (Active Motif Cat#39133): Dot blot analysis was used to confirm the specificity of Histone H3K27ac antibody.
https://www.activemotif.com/catalog/details/39133/histone-h3-acetyl-lys27-antibody-pab

Rabbit anti-H3K79me2 (Abcam Cat#ab3594): the manufacturer has validated the antibody specificity by western blot and by peptide array analysis. The manufacturer states: "ab3594 detects a 17 kDa band in single lane Western Blot. Peptide inhibition in Western Blot hasn't been processed. Modification specificity is determined by Peptide Array. ab3594 binds strongly to the Histone H3 dii methyl K79. In Peptide Array ab3594 also partially binds to mono methyl K79 and tri methyl K79 peptides".
https://www.abcam.com/en-it/products/primary-antibodies/histone-h3-di-methyl-k79-antibody-chip-grade-ab3594

Rabbit anti-H4K20me3 (Abcam, Cat#ab9053): the manufacturer has validated the antibody specificity by western blot. The manufacturer states: "ab9053 is specific for Histone H4 (tri-methyl K20). This is illustrated in lane 5 where the activity of ab9053 is specifically blocked by the addition of the immunizing peptide (ab17567)".
https://www.abcam.com/en-it/products/primary-antibodies/histone-h4-tri-methyl-k20-antibody-chip-grade-ab9053#all

Eukaryotic cell lines

Policy information about cell lines and Sex and Gender in Research

Cell line source(s)	Wildtype mouse embryonic stem cells (mESCs) were derived freshly (mixed 129/B6, XY) by the EMBL Rome Gene editing and Embryology facility. Catalytic-mutant Mll2 (Mll2CM/CM) and Setd2-/- homozygous ESCs were freshly derived from heterozygous FVB crosses carrying either an Mll2 Y2602A or a Setd2 KO allele.
Authentication	mESCs were authenticated by robust expression of pluripotency markers, morphology and by chimera formation and contribution to all embryonic tissues in vivo of the parental line (Carlini et al. 2022).
Mycoplasma contamination	mESCs were routinely tested for mycoplasma contamination by an independent commercial service using a highly-sensitive quantitative (q)PCR, with negative result each time.
Commonly misidentified lines (See ICLAC register)	N/A

ChIP-seq

Data deposition

☒ Confirm that both raw and final processed data have been deposited in a public database such as GEO.

☒ Confirm that you have deposited or provided access to graph files (e.g. BED files) for the called peaks.

Data access links *May remain private before publication.*	accession codes E-MTAB-12101, E-MTAB-13466, E-MTAB-13467, E-MTAB-13468.
Files in database submission	fastq
Genome browser session (e.g. UCSC)	No longer applicable.

Methodology

Replicates	3 replicates in each treatment setting for ChIP-seq and RNA-seq.
Sequencing depth	Libraries were sequenced on the Nextseq Illumina sequencing system (paired-end 40 sequencing).
Antibodies	Rabbit anti-H3K4me3 (Diagenode Cat#C15410003), Rabbit anti-H3K27me3 (Millipore Cat#07-449), Rabbit anti-H3K27ac (Active Motif Cat#39133)
Peak calling parameters	Analysis of the mapped sequences was performed using seqmonk software (Babraham bioinformatics, v1.47.0) by enrichment quantification of the normalised reads. To identify promoters with H3K4me3 change in Mll2 CM/CM, a 1kb window centered on the TSS was quantified amongst replicates and a normalised log fold-change (FC) filter applied between samples. Metaplots over genomic features were constructed by quantifying 100bp bins centered on the features of interest and normalised cumulative enrichments plotted.
Data quality	Raw Fastq sequences were trimmed to remove adaptors with TrimGalore (v0.4.3.1, -phred33 --quality 20 --stringency 1 -e 0.1 --

Data quality	length 20), quality checked and aligned to the mouse mm10 genome with the inserted mCherry reporter using Bowtie2 (v2.3.4.2, -I 50 -X 800 --fr -N 0 -L 22 -i 'S,1,1.15' --n-ceil 'L,0,0.15' --dpad 15 --gbar 4 --end-to-end -- score-min 'L,-0.6,-0.6').
Software	Seqmonk v1.46.0 Babraham bioinformatics https://www.bioinformatics.babraham.ac.uk/projects/seqmonk/<br>DESeq2 Love et al. 2014 https://bioconductor.org/packages/release/bioc/html/DESeq2.html<br>RNA Star Dobin et al. 2013 https://github.com/alexdobin/STAR<br>Trim Galore Krueger F. 2015 http://www.bioinformatics.babraham.ac.uk/projects/trim_galore/<br>FastQC Andrews S. 2010 https://www.bioinformatics.babraham.ac.uk/projects/fastqc/<br>FilterBAM Barnett et al. 2011 https://github.com/hammerlab/filter-bam<br>MarkDuplicates Tim Fennell https://broadinstitute.github.io/picard/<br>Bowtie2 Langmead and Salzberg 2012 https://github.com/BenLangmead/bowtie2

Flow Cytometry

Plots

Confirm that:

☒ The axis labels state the marker and fluorochrome used (e.g. CD4-FITC).

☒ The axis scales are clearly visible. Include numbers along axes only for bottom left plot of group (a 'group' is an analysis of identical markers).

☒ All plots are contour plots with outliers or pseudocolor plots.

☒ A numerical value for number of cells or percentage (with statistics) is provided.

Methodology

Sample preparation	Cells were washed with PBS and gently dissociated into single-cell suspension using TrypLE, followed by resuspension in FACS buffer comprised of PBS with 1% FBS, and filtered through a 40µm cell strainer (BD, cup-Filcons #340632). A FACS Aria III (Becton Dickinson) or Attune NxT Flow Cytometer (Thermo Fisher Scientific) were used for sorting or analysis, respectively.
Instrument	FACS Aria III (Becton Dickinson) or Attune NxT Flow Cytometer (Thermo Fisher Scientific)
Software	Flow cytometry data analysis was performed with FlowJo v10.5.3 (Tree Star, Inc.). Statistical analysis of flow cytometry data was performed using appropriate strategies in Prism GraphPad statistical software (v8.4.3).
Cell population abundance	For CUT&RUN experiments, from 1×105 to 1×106 cells (depending on the selected antibody) were flow sorted. For RNA extraction, 200.000 cells were flow sorted for each sample. A 4-Way Purity Precision Mode was used. A small portion of each sample was run again after each sort to verify that the intended cell population had been collected.
Gating strategy	Forward and side scatter density plots were used to distinguish between live cells and dead cells/debris. A side scatter height (SSC-H) vs side scatter area (SSC-A) plot was then used to exclude doublets. Next, two parameter density plots in which the X axis displays GFP fluorescence and the Y axis displays BFP fluorescence were used to gate for cells expressing all epigenetic editing components (GFP+; BFP+). Gates were pre-determined by running appropriate controls including GFP only and BFP only expressing cells and double negative cells (GFP-;BFP-). Gating strategy is also described in Extended data figure1.

☒ Tick this box to confirm that a figure exemplifying the gating strategy is provided in the Supplementary Information.
